# Retino-hypothalamic regulation of light-induced murine sleep

**DOI:** 10.3389/fnsys.2014.00135

**Published:** 2014-08-04

**Authors:** Fanuel Muindi, Jamie M. Zeitzer, Horace Craig Heller

**Affiliations:** ^1^Department of Biology, Stanford UniversityStanford, CA, USA; ^2^Department of Brain and Cognitive Sciences, Massachusetts Institute of TechnologyCambridge, MA, USA; ^3^Department of Psychiatry and Behavioral Sciences, Stanford UniversityStanford, CA, USA; ^4^Mental Illness Research, Education and Clinical Center, VA Palo Alto Health Care SystemPalo Alto, CA, USA

**Keywords:** melanopsin, photoreception, nocturnal, diurnal, suprachiasmatic nucleus

## Abstract

The temporal organization of sleep is regulated by an interaction between the circadian clock and homeostatic processes. Light indirectly modulates sleep through its ability to phase shift and entrain the circadian clock. Light can also exert a direct, circadian-independent effect on sleep. For example, acute exposure to light promotes sleep in nocturnal animals and wake in diurnal animals. The mechanisms whereby light directly influences sleep and arousal are not well understood. In this review, we discuss the direct effect of light on sleep at the level of the retina and hypothalamus in rodents. We review murine data from recent publications showing the roles of rod-, cone- and melanopsin-based photoreception on the initiation and maintenance of light-induced sleep. We also present hypotheses about hypothalamic mechanisms that have been advanced to explain the acute control of sleep by light. Specifically, we review recent studies assessing the roles of the ventrolateral preoptic area (VLPO) and the suprachiasmatic nucleus (SCN). We also discuss how light might differentially promote sleep and arousal in nocturnal and diurnal animals respectively. Lastly, we suggest new avenues for research on this topic which is still in its early stages.

## Introduction

The use of light for image-forming vision is critical in sighted animals as it is used to both detect and distinguish objects in the surrounding environment. In addition to its role in image-formation, light also exerts direct effects on physiology and behavior. These non-image forming processes include synchronization (entrainment) of circadian rhythms (Nelson and Takahashi, 1991), suppression of melatonin production (Klein and Weller, 1972; Lewy et al., 1980), modulation of the pupillary light reflex (Lucas et al., 2001, 2003), enhancement of cognition (Chellappa et al., 2011) and alertness in humans (Cajochen et al., 2000), and the acute induction of sleep in nocturnal animals (Alfoldi et al., 1991; Borbély et al., 1975; Borbély, 1976). The two-process model of sleep regulation has been used extensively to explain the timing of sleep. The model posits that sleep timing is a result of the combined influence of the homeostatic sleep process (Process S) and the circadian process (Process C; Borbély, 1982; Daan et al., 1984). As such, prolonged wakefulness increases the homeostatic sleep drive whereas the circadian process controls the division of sleep across the light and dark cycle. The circadian process is strongly influenced by light whereas the homeostatic sleep process is a function of prior sleep history. In nocturnal animals, the effects of light have been studied mostly in the context of circadian biology with a strong emphasis on the effects and mechanisms involved in the control of circadian rhythms by light (Golombek and Rosenstein, 2010). In contrast, the direct acute effects and mechanisms specifically involved in the modulation of sleep by light remain less well understood.

The discovery of melanopsin (Provencio et al., 2000), a photoreceptive molecule found in the mammalian retina, has played a crucial role in advancing our understanding of the input pathways modulating the acute effects of light on sleep and several other non-image forming processes (Panda et al., 2003). Approximately 1–2% of the retinal ganglion cells (RGCs) in the mouse retina express melanopsin and are intrinsically photosensitive (Hattar et al., 2002; Berson et al., 2010). They consist of several subtypes that are morphologically and physiologically distinct (Schmidt et al., 2011). The melanopsin expressing RGCs also receive light information arising from the rod and cone photoreceptors and together, the rods, cones, and melanopsin expressing RGCs account for all known light detection in the mouse retina (Hattar et al., 2003; Perez-Leon et al., 2006).

While melanopsin containing retinal ganglia have a clear and important role for the circadian system (Ruby et al., 2002), their role in mediating the photic induction and maintenance of sleep continue to emerge. A relatively small number of studies have assessed the separate roles of rod, cone and melanopsin photoreceptors in the regulation of sleep. The mechanisms downstream of the retina are similarly not well understood. This review discusses our current understanding of both the effects and mechanisms involved in the acute induction of sleep by light in mice at the level of the retina and the brain. We also discuss the how light might differentially promote sleep and arousal in nocturnal and diurnal animals respectively. Lastly, we suggest new avenues for research on this topic which is still in its early stages.

## Photic induction and maintenance of sleep

Many early studies examining the relationship between light and its capacity to induce sleep used short light-dark cycles of various durations (Borbély et al., 1975; Borbély, 1976; Alfoldi et al., 1991; Benca et al., 1998). These short light-dark cycles involve exposure to light for durations ranging from minutes to several hours. This approach is sensible as it allows the sampling of light effects on sleep across circadian phases. These data reveal that modulation of both non-rapid eye movement sleep (NREMS) and rapid eye movement sleep (REMS) are under circadian control such that NREMS induction is more pronounced early in the dark phase whereas REMS induction is more prominent during the subjective light phase (Borbély, 1978). However, the repeated pulsing of light makes the assessment of the acute effects and mechanisms involved difficult as the effects of light given at any given hour are likely to influence the effects of subsequent light pulses on sleep and wake. To solve this problem, recent studies (Table 1) have primarily used individual light pulses as short as a few milliseconds to more effectively assess the temporal dynamics of sleep initiation and maintenance by light. Moreover, such an approach has allowed a more mechanistic investigation of the pathways that may be involved.

**Table 1:** **An overview of studies examining the role of melanopsin in the acute induction of sleep**.

**Authors**	**Light Pulse Parameters Used**				**Observations**
	**Duration**	**Intensity**	**Time of Day**	**Spectrum**	
Lupi et al. (2008)	1 h	200 μW	ZT16	Broad	Normal sleep induction in rd/rd cl mice No sleep response in MKO mice
Altimus et al. (2008)	3 h	1000 lux	ZT14	Broad	Sleep induced during the initial 30-min in both MKO and rod/cone less mice
Tsai et al. (2009)	1 h 1:1 LD Schedule	~80–90 lm/W	ZT15 and across the circadian cycle	Broad	MKO mice fail to respond to light pulses during the dark period (ZT15-23) Reduced delta power in MKO mice during the dark period
van Oosterhout et al. (2012)	1 h	12.9 log quanta/cm^2^/s	ZT16	Ultraviolet	UV light induces sleep in both WT and MKO mice with equal e_cacy
Muindi et al. (2013)	Various 15 min-6 h	0.2–200 μW	ZT13	Broad	Sleep is induced within 10 min in both WT and MKO mice Sleep is initially induced during the 1st hour but is not maintained in MKO mice

*A summary of the studies to date that have assessed the role of rod, cone and melanopsin photoreception in the acute induction of sleep. Note that it would be useful to have a single unit of light measurement so as to improve comparability of light sources used across studies. However, there is currently no agreement as to which intensity unit should be used as a common unit. In the context of this table, these units are not interconvertible. Abbreviations: Melanopsin Knockout, MKO; Wild-type, WT; Light-Dark, LD; Zeitgeber, ZT; Rodless/Coneless, rd/rd cl*.

### Role of melanopsin in sleep maintenance

Lupi et al. (2008) first described the role of melanopsin signaling in the acute induction of sleep by exposing mice lacking melanopsin or lacking both rods and cones to a 1-h saturating light pulse during the dark phase (ZT16-17). Saturating light was able to induce sleep in mice lacking both rods and cones, but failed to do so in melanopsin knockout mice (MKO). The initial suggestion was that sleep induction is predominantly or exclusively mediated by melanopsin photoreception. However, further experiments from Altimus et al. (2008) suggested that rod/cone and melanopsin photoreception are both necessary to mediate the full effects of light on sleep. One important difference between the studies is in the duration of light exposure utilized. Instead of using a 1-h light pulse, Altimus et al. exposed both rod/cone-less and MKO mice to a 3-h light pulse beginning at ZT14. This allowed them to assess sleep over a longer time frame. In contrast to the findings by Lupi et al. (2008), a time course analysis showed an initial induction of sleep by light in the first 30 min in both genotypes. However, the response was not maintained for the remainder of the 3-h light pulse in both genotypes. This apparent lack of sleep maintenance would have been difficult to observe in the study by Lupi et al. (2008) as only a 1-h pulse was used in a relatively small number of animals. It is also important to note that preceding sleep amounts before the light pulse were not shown in any of the genotypes across both studies. The lack of such data may also have contributed to the inconsistency of the results between the studies, particularly concerning the role of rods/cones and melanopsin photoreceptors in sleep induction. Despite the differences, these studies collectively implicate an important role for melanopsin photoreception during the induction of sleep by light.

Since these initial reports, three additional studies have addressed the role of melanopsin photoreceptors in the induction of sleep by light (Table 1). Tsai et al. (2009) examined the role of melanopsin in mediating the effects of light on the interaction between the circadian and sleep homeostatic systems. In that study, the authors extensively characterized the acute effects of light in MKO mice under various light-dark paradigms. They reported a circadian influence over the light-induced sleep response in that it was mostly present in the early phases of the light and dark periods in MKO mice. In fact, light-induced sleep response was absent for much of the dark period after ZT15 and was attenuated during the light period. These results confirmed observations by both Lupi et al. (2008) and Altimus et al. (2008) with respect to the differences in the sleep response due to the time of day. However, an important observation by Tsai et al. was the attenuation of slow wave activity in MKO mice during the dark period prompting the authors to suggest a role for melanopsin in sleep homeostasis. This claim was further substantiated in the same study by the observation of an attenuated increase in slow wave activity following 6-h of sleep deprivation during the light period in MKO mice in comparison to wild-type (WT) mice. These findings suggest a complex interaction between melanopsin and sleep homeostasis within the current framework of the two-process model of sleep regulation. It is likely that the absence of melanopsin causes changes in the interaction between the circadian and homeostatic processes. Further studies are required to better understand the contribution of different photoreceptors in the two-process model of sleep regulation.

The second study by van Oosterhout et al. (2012) further refined our perspective concerning the role of rod/cone and melanopsin photoreception in light-induced sleep through the use of narrow-band spectrum light in the ultraviolet (UV) range. In contrast to the previous studies in which the effects of light on sleep were assessed only with full-band spectrum light, van Oosterhout et al. showed that a 1-h exposure to UV light at ZT16 can induce sleep in both WT and MKO mice with equal efficiency thus suggesting that the UV light induction of sleep occurs independent of melanopsin. However, the light exposures were limited to 1-h, at a single circadian time, and at a single intensity. As such, we only have a limited view of the effects of UV light on sleep and its interaction with the circadian system. Nevertheless, the result further supports the idea that the transmission of photic information vis-á-vis sleep induction is mediated by the combined signaling of the rods, cones and melanopsin photoreceptors. A recent study from our lab has further supported the claim that melanopsin photoreceptors play a critical role in the maintenance of light induced sleep (Muindi et al., 2013). Following exposure to saturating light, we observed no differences in total sleep amounts between WT and MKO mice during the first hour of light exposure. We also found that sleep latency was similar between the genotypes, with sleep beginning 10–15 min after light pulse onset. These data provide support for the initiation of light-induced sleep not being dependent upon functional melanopsin photoreceptors. The initial evidence for this idea was reported by Mrosovsky and Hattar (Mrosovsky and Hattar, 2003). In their study, the suppression of locomotor activity during a 3-h light pulse was initially comparable between WT and MKO mice. However, the suppressive action of light on activity in MKO mice gradually diminished over the next hour such that they returned to baseline locomotor activity levels by 100 min. By using a 6-h continuous light pulse, our study further showed that the ability to maintain sleep became highly variable in WT mice after 3-h. Lastly, we found an increase in both locomotor activity and a decrease in sleep in WT mice 1-h post pulse—a result not observed in the MKO mice. We suggest that the decrease in photically induced sleep is part of compensatory changes that return sleep to baseline levels (i.e., the response is homeostatically regulated). Support for this notion is further substantiated by the recent finding in mice where 24-h total sleep remains the same across a wide range of short LD cycles (Deboer et al., 2007). Similarly, total sleep during the entire 12-h dark period does not change when compared to baseline in both WT and MKO mice in our study. We propose that the compensatory changes observed almost immediately after light pulses of several durations work to maintain 24-h total sleep amounts constant.

**Figure 1:**
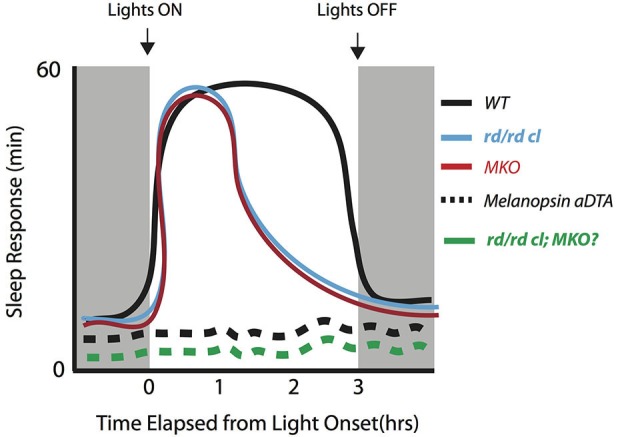
**Initiation and maintenance of sleep across different genotypes early in the dark period to a bright saturating light pulse**. The induction of sleep can be maintained for at least 3-h early in the dark period in WT mice (black line). In the absence of melanopsin (MKO) or rod-cone photoreception (*rd/rd cl*), there is an initial increase in sleep but lack of maintenance for the remainder of the light pulse in both genotypes (Altimus et al., 2008; Muindi et al., 2013; red and blue lines). When the melanopsin expressing RGCs are selectively ablated by the expression via the expression diphtheria toxin-A (aDTA) in the melanopsin locus (Altimus et al., 2008) the light-induced sleep response is completely abolished (black dotted line). This suggests that these cells serve as an exclusive pathway for mediating the acute effects of light. The sleep response in the triple knockout (*rd/rd cl/*MKO) is currently not known, but is likely to mimic the results from the melanopsin aDTA mice (green dotted line).

Altogether, studies thus far suggest that rod, cone and melanopsin photoreception are each involved in the acute modulation of light-induced sleep in mice during the dark phase. However, they may differentially contribute to the initiation vs. the maintenance of sleep. Current data support the view that melanopsin photoreception is necessary for the maintenance of light induced sleep (Figure 1). In the absence of melanopsin, rod-cone photoreception is sufficient to initiate sleep in response to a light stimulus but is unable to sustain light-induced sleep. However, the involvement of melanopsin photoreception during the initiation of sleep cannot be ruled out. More studies are required to specifically assess the influence of rod-cone photoreception in the acute induction of sleep by light across the circadian cycle. These studies will need to employ different light intensities across durations of light exposure lasting at least 3-h in mice lacking rod-cone and/or melanopsin photoreception. Such experiments will provide additional insights into the involvement of the photoreceptors across different light exposure paradigms that induce sleep in rodents. Emphasis will need to be placed on the periods before and after the light pulses in order to provide a full picture of the response. Altogether, the data thus far suggest a critical role for melanopsin in maintaining light induced sleep for extended periods of time during the dark period.

### Sleep induction with millisecond light flashes

Recent evidence has suggested that continuous light is not necessary to induce sleep in rodents. Studies using millisecond light flashes have demonstrated that continuous light is not required for either the suppression of wheel running locomotor activity or the initiation of sleep (Vidal and Morin, 2007; Morin and Studholme, 2009; Studholme et al., 2013). These data reveal that as few as 10 2-ms flashes delivered over 5 min are sufficient to rapidly suppress mouse locomotor activity and initiate sleep within 10 min (Studholme et al., 2013). The observed sleep latency is consistent with sleep onset during longer light exposures as shown by others (Lupi et al., 2008; Muindi et al., 2013). Surprisingly, the initiated sleep by the light flashes is maintained for an additional 20 min without further light exposure before returning to baseline sleep amounts (Morin and Studholme, 2009; Studholme et al., 2013). This phenomenon has also been observed in hamsters where brief light exposure is able to suppress wheel running for a similar amount time after the light pulse has ended (Redlin and Mrosovsky, 1999). Thus, it seems that light information is likely integrated both at the level of the eye and likely in other areas across the brain to drive a relatively rapid and sustained sleep response for some time after an acute exposure to millisecond light flashes. As it is now clear that continuous light is not necessary for sleep induction in mice, a few questions need to be addressed moving forward.

First, what is the integration capacity of the mouse non-image forming visual system for sleep initiation and maintenance? A simple approach is to conduct a parametric investigation with different light flash patterns to determine how the light-induced sleep varies with flash number and inter-flash interval similar to that conducted by Morin and Studholme (2009) for locomotor activity suppression. Such an approach will also prove useful in better assessing the temporal changes in NREMS and REMS during and after the flashes. Second, can millisecond light flashes sustain the sleep response in the absence of melanopsin photoreception? If the rod/cone system adapts and fails to sustain the response to light, as suggested by Altimus et al. (2008), it could be the case that the darkness between flashes of light is necessary to provide the required recovery time window during the dark periods. This assertion may be possible as rod/cone photoreceptors exhibit: (1) a relatively fast recovery time from a saturating flash stimulus (Lyubarsky and Pugh, 1996); and (2) rods are mostly nonfunctional in continuous saturating light conditions due to bleaching (Green, 1971). Investigating these questions will allow us to better understand the relative roles of rod/cone and melanopsin photoreception in the induction of sleep by light. Altogether, sleep induction by millisecond light flashes provides an additional opportunity to better understand the integration of photic information across the neural circuits involved in the induction of sleep in mice.

## Neurobiology of the photic induction of sleep

The projections of the melanopsin containing RGCs are well described (Hannibal and Fahrenkrug, 2004; Hattar et al., 2006). Briefly, these RGCs project to several areas in the brain. However, the main projections of the melanopsin expressing RGCs within the retino-hypothalamic tract (RHT) are to the following regions: hypothalamus, lateral geniculate complex (LGC), olivary pretectal nucleus (OPN), and superior colliculus (SC). In the hypothalamus, the suprachiasmatic nucleus (SCN), subparaventricular zone (SPZ), ventral lateral preoptic area (VLPO) and lateral hypothalamus (LH) all receive RHT innervation. The former two areas (SCN, SPZ) are crucial in the circadian timing system while the latter two areas (VLPO, LH) are important modulators of sleep and wake. Despite the characterization of these neural pathways, the mechanisms involved in the processing of photic information in areas innervated by the RHT during the induction of sleep by light are not well understood. However, some suggestions can be drawn from existing evidence.

### Suprachiasmatic nucleus

The SCN is the master regulator of circadian rhythms in mammals and is densely innervated by the RHT (Moore and Lenn, 1972; Stephan and Zucker, 1972). It controls an elaborate network of hypothalamic circuits involved in a number of physiological and behavioral rhythms, including sleep and wake. With respect to the control of sleep by the SCN, three models have been suggested (Ibuka and Kawamura, 1975; Mistlberger et al., 1983; Edgar et al., 1993; Baker et al., 2005). Evidence for each of the models is extensively reviewed by Mistlberger (2005). Briefly, the first model suggests the SCN actively promotes arousal during the daily active phase. The second suggests the SCN actively promotes sleep during the daily rest phase. The third suggests the SCN actively promotes arousal during the active phase and sleep during the rest phase. Much of what we know about the direct effects of the SCN on sleep and wake in rodents comes from lesion studies and knockout of core clock genes. Due to the lack of spatial and the much desired temporal control of the SCN, such approaches alone have made it difficult to fully understand the direct effects of SCN neural activity on sleep. The electrophysiological characteristics of the SCN and its response to photic stimuli have been well described. In nocturnal rodents, SCN neurons have elevated firing during the light phase (sleep predominant portion of the daily cycle) and lower firing during the dark phase (Meijer et al., 1998). This pattern of firing due to ocular light exposure can be simulated by electrical stimulation of the RHT (Shibata et al., 1984) or direct application of RHT neurotransmitter, glutamate, to the SCN (Schmahl and Bohmer, 1997). Light-modulation of SCN firing is both dose- and time-of-day-dependent, following the behavioral effects of light on circadian rhythms (Meijer et al., 1998). Light pulses induce a fast, transient increase in SCN firing rate that is followed by a steady state response. A rapid decrease in activity below baseline level is often observed after light is turned off before returning to baseline after a few minutes (Meijer et al., 1998).

With respect to SCN electrical activity and sleep, the study by Deboer et al. (2003) is the only report thus far to have combined long-term recordings of SCN electrical activity in freely moving animals while simultaneously recording sleep. The authors report that SCN activity is elevated during the transition from NREMS to REMS, and from NREMS to wake. Conversely, a reduction in activity is observed during the transition from wake to NREMS. By depriving the animal of either NREMS or REMS, the authors showed that the SCN neurons do not show the observed changes in electrical activity prompting the conclusion that sleep state information is able to alter SCN electrical activity. Whether or not the SCN itself is sufficient to influence sleep state transitions is not clear. This may be possible partly because the SCN consists of GABA-expressing neurons sending both monosynaptic and multisynaptic connections to hypothalamic nuclei implicated in sleep and wake (Mistlberger, 2005). Although these projections are sparse, it is conceivable that the influence of the SCN on sleep state transitions may come from the direct and indirect modulation of sleep and wake circuits. One area known to receive direct but sparse input from the SCN is the wake promoting tuberomamillary nucleus (TMN; Abrahamson and Moore, 2001; Deurveilher and Semba, 2005). Interestingly, histamine levels in some brain regions have been shown to cycle across the light and dark cycles (Orr and Quay, 1975; Tuomisto and Tuomisto, 1982; Rozov et al., 2014). This suggests that some circadian control of the histaminergic system is likely to be present for select areas in the brain. As mentioned earlier, a major limiting step in teasing apart the relationship between the SCN and sleep-wake states has been the inability to selectively modulate SCN activity. Future studies may improve our understanding of the relationship between the changes in SCN activity and the transitions in sleep states by turning to new targeting technologies such as optogenetics and pharmacogenetics in combination with *in vivo* electrical recordings. These approaches provide the spatial and the much desired temporal control required for effectively assessing the link between SCN activity and sleep transitions.

### Ventrolateral preoptic area and other sleep-wake related areas

The VLPO consists of a dense hub of sleep-active, GABA-expressing neurons that project to several wake promoting areas (Steininger et al., 2001). The VLPO is sparsely innervated by the RHT (Lu et al., 1999). FOS protein expression, a marker of neuronal activity, increases in the VLPO with exposure to light and is significantly attenuated in MKO mice (Lupi et al., 2008; Tsai et al., 2009). As such, one hypothesis is that photic activation of the VLPO shifts the balance towards sleep by increasing the inhibition of wake promoting circuits. No studies have thus far specifically assessed whether the VLPO is necessary during the induction of sleep by light. As such, the degree to which the VLPO is involved during the response is not known. Complicating matters are the additional projections of the RHT to other brain areas involved in the regulation of sleep and wake. These areas include the LH, superior colliculus-pretectum (SC-PT), SPZ, and basal forebrain (BF; Youngstrom et al., 1991; Leak and Moore, 1997; Hattar et al., 2006). The LH contains both the wake promoting hypocretin (HCRT) cells and the putative sleep-promoting melanin concentrating hormone (MCH) producing cells (Gerashchenko and Shiromani, 2004). The SC-PT processes visual information but has also been implicated in the photic regulation of sleep in albino rats (Miller et al., 1998). On the other hand, the direct roles of the SPZ and BF in the acute induction of sleep by light remain unclear. Recently, ghrelin-immunopositive neurons have been identified within the SPZ (Horvath et al., 2012). The study showed that the ghrelin labeled neurons are innervated by the SCN and the lateral geniculate nucleus (LGN), a visual center, and project to the LH. It is then likely that these neurons are important mediators of circadian and visual information to cells in the LH. The dorsomedial hypothalamus (DMH), a region innervated by the SPZ (Chou et al., 2003) is also likely to play an important role during photosomnolence. The DMH sends inhibitory GABAergic projections to the VLPO, and excitatory glutamatergic projections to the hypocretin- and melanin-concentrating hormone-producing neurons in the LH and is an important integrator of SCN information (Chou et al., 2003). Altogether, it is likely that these areas are all involved in the photic regulation of sleep. The extent to which each area is involved is currently not known.

## Nocturnality and diurnality: single or multiple switches?

Nocturnal animals are mostly active during the dark period and spend a significant portion of the light period sleeping. This behavior contrasts that in diurnal animals where the timing of behavior is reversed. In contrast to the nocturnal mouse, exposure of acute light pulses in the diurnal Nile grass rat (*Arvicanthis niloticus*) has been shown to increase locomotor activity during the dark period (rest period; Shuboni et al., 2012). In humans, light-induced arousal is more prominent at high intensities (Cajochen et al., 2000) and at short wavelengths (Revell et al., 2006; Rahman et al., 2014) as assessed by subjective and objective measures electroencephalography (EEG) of arousal. Interestingly, an increase in arousal has also been documented in humans after exposure to brief flashes of light (2-ms at 473 lux) occurring once per minute for a period of 60 min during the dark period (Zeitzer et al., 2011). This differential response between nocturnal and diurnal species raises an important question regarding the neural mechanisms involved. One hypothesis suggests that the differential responses to light are not controlled by pathways communicating light information from the retina to the SCN, but rather by downstream pathways from the SCN. This idea comes from data showing similar profiles in SCN electrical activity rhythms (Inouye and Kawamura, 1979; Sato and Kawamura, 1984; Kurumiya and Kawamura, 1988; Meijer et al., 1998) and core clock gene expression (Mrosovsky et al., 2001; Lincoln et al., 2002; Caldelas et al., 2003) in both nocturnal and diurnal animals. Moreover, neural organization of the circadian system in diurnal animals (Goel et al., 1999; Schwartz et al., 2011) resembles that of the widely studied nocturnal rodents (Mistlberger, 2005). Presumably, diurnal and nocturnal differences are due to functional changes in one or a few areas within the circadian neural network. One area that has shown a fundamental difference is the SPZ. Like in the nocturnal laboratory rats, the ventral portion of the SPZ (vSPZ) in the diurnal Nile grass rat receives dense innervation from the SCN (Schwartz et al., 2011). The vSPZ also projects to nearly all of the same areas as the SCN in both nocturnal and diurnal rodents (Schwartz et al., 2009). However, in the diurnal Siberian chipmunk, multi-unit activity dorsal to the SCN area (close to the vSPZ) has been shown to increase during the light period and drop during the dark period (Sato and Kawamura, 1984). This relationship was reversed in the nocturnal laboratory rats with vSPZ activity peaking during the dark period (Inouye and Kawamura, 1979). In contrast, FOS (a marker of neural activity) in the vSPZ of the diurnal Nile grass rat has been reported to be higher during dark period and about 180° out of phase with activity in the SCN (Schwartz et al., 2004). Lesions of the vSPZ in the nocturnal rat abolish sleep (Lu et al., 2001) and locomotor rhythms (Lu et al., 2001; Abrahamson and Moore, 2006). A similar result has been observed in the diurnal Nile grass rat where vSPZ lesioned animals display unstable and weakened rhythms in locomotor activity (Schwartz et al., 2009). The study also reported crepuscular bouts of activity in all of the animals with vSPZ lesions. Together, the data suggest that the vSPZ is one of the principal areas involved in the regulation of nocturnality and diurnality.

Are there other areas? Additional data provide some clues. A recent study by Gall et al. (2013) suggested that the intergeniculate leaflet (IGL) nucleus of the thalamus is also involved in regulating the difference between nocturnal and diurnal species. This region is innervated by the RHT and is also reciprocally connected with the SCN (Morin and Allen, 2006). In the study by Gall et al. (2013), lesions of the IGL in the diurnal Nile grass rat led to a significant increase in nighttime activity. When the acute effects of light were assessed, light pulses increased activity in intact Nile grass rats (positive masking), but caused a suppression (negative masking) in IGL lesioned rats (Gall et al., 2013). On the other hand, IGL lesions in the nocturnal Syrian hamster cause an enhancement in negative masking (Redlin et al., 1999). Taken together, it is likely that the IGL and the vSPZ work together to regulate the differential behavioral responses to light in between nocturnal and diurnal animals. However, questions of how these areas acutely modulate the differential effects of light on arousal in nocturnal and diurnal animals require further investigation.

Although it is far simpler to have a single switch, it is possible that multiple pathways are involved in determining chronotype. Consistent with this idea, other lines of evidence have suggested that retinal pathways are also involved in determining chronotype. A targeted double knockout of Rpe65 (a key enzyme used in retinal chromophore recycling) and melanopsin in mice has been shown to cause a switch to diurnal activity patterns in 80% of the animals (Doyle et al., 2006). The switch was accompanied with a reversal in *Per2* expression within the SCN and in the negative masking effect of light (Doyle et al., 2008). The reversal of *Per2* expression in the double knockouts is interesting because it is opposite to what has been described in both nocturnal and diurnal animals (Mrosovsky et al., 2001; Caldelas et al., 2003). The authors suggest that the reversal is likely the result of upstream changes in the retina in mice lacking Rpe65 and melanopsin (Doyle et al., 2008). Additional data from the same study also showed that a switch to diurnal pattern of activity was possible in WT mice when they were housed in dim light-dark cycles below the thresholds of cones and melanopsin (Doyle et al., 2008). These results prompted the authors to conclude that retinal pathways themselves are sufficient to influence an organism’s temporal niche. Additional studies are, however, required to further characterize the impact of the retinal changes to downstream brain areas innervated by the RHT. It is conceivable that changes to downstream brain areas could also have contributed to the reversal. Complicating matters further is the ability to switch between nocturnal and diurnal patterns in several species (Hut et al., 2012). Some level of plasticity likely exists within the pathways to enable animals to quickly respond and adapt to changes in their environments. Different photoperiods have recently been shown to drive changes in neurotransmitter expression in populations of interneurons within the hypothalamus (Dulcis et al., 2013). Such changes may take place in animals that are able to switch between nocturnal and diurnal behavioral patterns. Taken together, the data thus far suggest that there may be more than one area modulating the reversal between nocturnal and diurnal animals. Whether a master switch exists remains unknown.

## Light-induced sleep: working model

**Figure 2:**
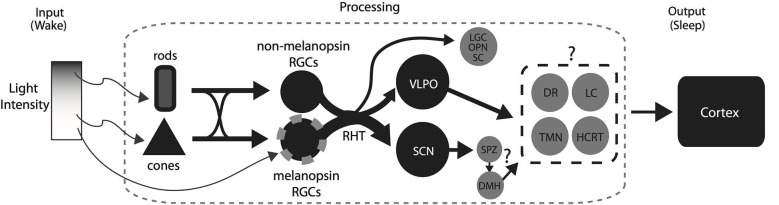
**A summary diagram showing the pathways and areas that may be involved in the direct modulation of sleep by light in nocturnal animals**. At low light intensities, rod photoreception relays light information to RGCs expressing melanopsin and RGCs not expressing melanopsin. Light activates both the SCN and the VLPO via the RHT and most likely causes the inhibition of the downstream wake promoting areas. At higher intensities, the rod-, cone-, and melanopsin photoreception work together to initiate and maintain the activation of the VLPO and SCN resulting in the maintenance of sleep during a light pulse early in the dark period. Similarly, the inhibition of the wake promoting areas by the VLPO and presumably the SCN via the SPZ and DMH would facilitate the promotion of sleep by light at high intensities. However, it is not known whether the inhibition is across some or all of the wake promoting areas. Abbreviations: Retinal Ganglion Cells, RGCs; Retino-hypothalamic tract, RHT; Suprachiasmatic Nucleus, SCN; Ventrolateral Preoptic Area, VLPO; Lateral Geniculate Complex, LGC; Olivary Pretectal Nucleus, OPN; Superior Colliculus, SC; Dorsomedial hypothalamus, DMH; Dorsal Raphe, DR; Locus coeruleus, LC; Tubero-mammillary nucleus, TMN; Hypocretin, HCRT.

The flip-flop switch model has been used extensively to highlight the control of behavioral state by the sleep and wake promoting systems. Briefly, the model proposes a mutual inhibition between the sleep and wake promoting systems which allow both rapid and stable transitions (Saper et al., 2010). It was recently suggested that light alters the balance of the flip-flop switch by the activation of the VLPO which in turn inhibits the wake promoting areas thus promoting sleep (Hubbard et al., 2013). Consistent with this idea, we have summarized our current understanding of the mechanisms involved in a simple model with some important additions (Figure 2). In the retina, rod-, cone-, and melanopsin-based photoreception work together to provide the necessary signaling for the initiation and maintenance of sleep by light. In the brain, the RHT activates both the SCN and the VLPO which presumably cause the suppression of the wake promoting systems resulting in the induction of sleep. However, a number of questions remain: (1) beyond its role as a circadian clock, does the SCN play a direct role in the initiation and maintenance of sleep by light?; (2) how do the changes in light intensity affect neuron activity in the VLPO and the DMH?; (3) what is the extent of inhibition in the wake promoting areas during the light exposure? Are they activated instead in a diurnal brain?; and (4) what is the sequence of activation and/or inhibition of sleep and wake promoting areas that result in the rapid initiation of light induced sleep in nocturnal rodents? Investigating these questions may be a worthwhile endeavor as they will unravel how the brain integrates the photic input from the retina to enable the rapid initiation and maintenance of sleep induced by light.

The suggestion that light initiates a sequence of activation and/or inhibition across a range of regions during the induction of sleep has also been suggested to exist for the suppression of pineal function, phase shifting, corticosterone release, and core temperature decrease (Morin, 2013). The proposed model suggests that light triggers a sequence of events within the SCN to initiate most of these events. With respect to the induction of sleep by light, it is likely that in addition to the intrinsic sequence of activation within the SCN, a more general sequence of activation and perhaps inhibition is triggered by light across multiple brain areas. Therefore, understanding the pattern of activation and/or inhibition during the initiation and maintenance of sleep by light is likely to yield important answers. The initial approach will require the simultaneous recording of neurons across both sleep and wake systems whilst examining their firing patterns during the light induced transition from wake to sleep. This is theoretically possible, but such chronic recordings in freely moving animals are likely to be a challenge due in large part to the proximity and depth of many of the areas of interest. To assess causality, such an approach will need to be combined with optogenetics or pharmacogenetics to assess both the necessity and sufficiency of some of the areas proposed to be involved. However, careful consideration must be given to the stimulation or inhibition of sleep and wake circuits at physiologically relevant times of day (Sidor and McClung, 2014). Traditional techniques such as lesions and pharmacology can also be used for areas not yet accessible by optogenetics or pharmacogenetics to provide additional insights into the direct regulation of sleep and wake by light. Altogether, the induction of sleep in nocturnal rodents by light presents an opportunity to systematically assess the circuits involved at the critical transition from wake to sleep.

## Summary

In this review, we discussed our current understanding of the neural circuits involved in the control of murine sleep by light at the level of the retina and the downstream brain areas. At the level of the retina, the rod, cone and melanopsin photoreceptors work together to drive a continuous light signal to the sleep promoting system in the brain (Figures 1 and 2). However, the extent to which each photoreceptor contributes to the rapid initiation and the maintenance by light will require further study. At the level of the brain, some or all of the wake promoting areas are inhibited by the activation of the VLPO and SCN in nocturnal animals, and we suggest the response in wake promoting areas is reversed in diurnal animals. Although densely innervated by the RHT and rapidly activated by light, the role of the SCN in the acute induction of sleep and arousal in nocturnal and diurnal animals respectively is not clear.

## Conflict of interest statement

The authors declare that the research was conducted in the absence of any commercial or financial relationships that could be construed as a potential conflict of interest.
